# Supplementary material for: Situating trade-offs: Stakeholder perspectives on overtreatment versus missed diagnosis in transition to Xpert MTB/RIF Ultra in Kenya and Swaziland
Source: PLoS One. 2020 Feb 19;15(2):e0228669. doi: 10.1371/journal.pone.0228669 (PMC7029953; doi:10.1371/journal.pone.0228669)
Supplement: S1 Appendix — (PDF) [file pone.0228669.s001.pdf]

S1 Appendix. Focus Group Discussion Questions

1. On a post-it, please write down challenges/successes that you have experienced in TB diagnosing using Gene Xpert in your setting (if you do not use Gene Xpert, any diagnostic tool)
2. Discuss each challenge/success in greater depth
    1. How do you expect this would change if you were to adopt Ultra?
3. How can Ultra be introduced into the current algorithm?
    1. How will you adopt Ultra
    2. How have TB tests been adopted in the past? Vs Now?
    3. Experiences with adoption and implementation of existing cartridge?
4. If trade off (sensitivity vs specificity) is not brought up naturally…
    1. "research shows that Ultra is more sensitive but less specific…what are your thoughts on this? Thoughts of over treating? Thoughts on under-diagnosing?
    2. Have you dealt with a similar trade off in the past?
    3. How will you deal with this trade off with Ultra?
