# Supplementary material for: Situating trade-offs: Stakeholder perspectives on overtreatment versus missed diagnosis in transition to Xpert MTB/RIF Ultra in Kenya and Swaziland
Source: PLoS One. 2020 Feb 19;15(2):e0228669. doi: 10.1371/journal.pone.0228669 (PMC7029953; doi:10.1371/journal.pone.0228669)
Supplement: S2 Appendix — (PDF) [file pone.0228669.s002.pdf]

S2 Appendix. Participant Responses to Success and Challenges in TB Diagnosis
Kenya
Challenges
Group 1:

- Difficulty convincing the patient to seek the clinicians help in case of positive results
- Dealing with patients who feel that the result must be positive
- Access and utilization of Gene Xpert is sub-optimal
- Cost of sample networking is still prohibitive in Xpert utilization; 100% partner supported
- Dealing with indeterminate Rif results
- Reduced number of clinically diagnosed cases
- Having discordance results in RS/RR i.e Xpert vs LPA/DST culture
- Repeat in case of invalid results
- Management of symptomatic presumed TB cases with Xpert negative still a challenge
- Invalid results i.e culture
- Contaminants
- Errors/Invalid results
- Errors beyond the technical staffs ability to troubleshoot
- Large pool of Xpert negative during AF not well managed
- Not universal yet, not accessible to all patients
- It was hoped that cased finding would increase significantly due to increased sensitivity but hopefully with even further utilization rates, we will see greater increase
- When does Xpert get negative after a duration of TB treatment still unknown
- Using Xpert as part of diagnostic algorithm (clinicians still trust their judgement more than a negative Xpert in cases of smear negative, Xpert negative TB)
- Poor timing before setting the next test
- Discordant results between Xpert and culture still cause a lot of indecision
- No reliable bedside test
- Diagnosis of EPTB; lack of standardized guidelines on use of Xpert in these cases
- Diagnosis in children
- Diagnosis in HIV positive individuals
- Supplies
- Interruption during power failure
- Sputum transport network; strengthen consistency → public private partnerships to strengthen referral system from the smaller formal and informal TB nondiagnostic health facilities e.g chemists, small clinics, informal providers; in order to intensify case finding
- Stock out of cartridges on the ground with facilities closing → lose out on MDR surveillance and universal access to drug susceptibility
- Nasopharyngeal and stool as alternative samples based on CDC research challenge to the program

- Logistics (commodity) supply chain still not well controlled → frequent stock outs of cartridges

Group 2:

- Numerous MDR cases coming up
- Low case finding
- Government not willing to publicize cartridge (GXP) stock out and provide solutions
- Shortage of cartridge that is frequent
- Health facilities far apart and most of them have no laboratories; patient travel far distance for diagnosis
- Lack awareness and availability of Gene Xpert in the rural health care workers
- Lack of sample referral system
- MOTTS being treated as TB
- Contamination of TB samples
- Discordant results between different tests
- Patients who are too ill to give sputum; they need Xray which is expensive
- Transportation of samples is a challenge in hard to reach areas
- Misdiagnosis of extra-pulmonary TB
- TB diagnosis in children is difficult since they cannot give sputum
- Its great that Kenya was early adopter of GXP MTB/RIF, I hope they will be more organized and prepared to implement and roll out Ultra

Group 3:

- Staff strikes affect effective diagnosis and patient management
- Stock outs is enough
- Sample transportation from peripheral labs in the private sector due to lack of support for transport
- Poor collection of samples leads to contaminated samples
- Shortage of cartridges
- Inadequate Xpert training in some countries
- Power supply shortage in some parts of the country
- Shortage of staff in most lab settings
- Stock outs
- Shipment of samples not in good condition
- Erratic supply of commodities
- Low utilization rate for Xpert

Successes

Group 1:

- Reduced number of clinically diagnosed cases
- Increased the number of bacteriologically confirmed cases
- Led to decreased attrition at DX compared to sputum microscopy

- Reduced DX cost for the patient in terms of repeated visits for sample collection, transport costs, time and loss of wages
- Increased TAT for TB DX and hopefully reduced transmission

Group 2:

- Successful surveillance of TB in Kenya 2016
- Its great that Kenya was early adopter of GXP MTB/RIF, I hope they will be more organized and prepared to implement and roll out Ultra
- Quick DX of RIF resistance
- Effective tests
- Timely and efficient reporting and results delivery to the customer due to GX alert

Group 3:

- Staff skills and competency
- Staff dedication and commitment
- Management support
- Shortage in treatment time of patients who show resistance to Rif
- TAT in TB diagnosis is achieved
- Serve the people of the republic of Kenya in diagnosing TB in a timely manner with the correct diagnosis
- Timely diagnosis of all TB suspects
- Accuracy of diagnosis
- Good management support
- Free TB management

Swaziland

Challenges

- Bacteriological diagnosis in children due to obtaining viable sputum samples, this leads to underdiagnoses of TB in this population
- Bacteriological diagnosis in PLHIV
- Expensive to maintain the Gene Xpert hardware
- Erratic power supply
- Stock out due to problems in the supply chain
- Error logs
- Running insufficient samples
- Limitation to detection of RIF resistance and not other anti-TB medication
- Obtaining EPTB samples
- Dealing with unlabeled specimen
