# Supplementary material for: Situating trade-offs: Stakeholder perspectives on overtreatment versus missed diagnosis in transition to Xpert MTB/RIF Ultra in Kenya and Swaziland
Source: PLoS One. 2020 Feb 19;15(2):e0228669. doi: 10.1371/journal.pone.0228669 (PMC7029953; doi:10.1371/journal.pone.0228669)
Supplement: Attachment — Submitted filename: Reviewer Comments 3.pdf [file pone.0228669.s003.pdf]

Reviewer #: This research article examines the perspectives of stakeholders on the trade-off between over-treatment and missed diagnosis captured during decision-making workshops on the transition from use of Xpert MTB/RIF to diagnose tuberculosis to Xpert MTB/RIF Ultra in Kenya and Swaziland.

The aim of the research was to examine the views and norms of multiple TB stakeholders on the trade-off between overtreatment versus under diagnosis of TB, and to understand the role qualitative research can play in engaging in-country stakeholders during the launch and roll-out of new TB diagnostics.

Positioning qualitative methodology within TB workshops provides a unique opportunity to identify perspectives and everyday experiences of key stakeholders and implementers and to contextualize the introduction of a diagnostic test and the interpretation of a particular trade-off within the realities of these everyday experiences.

Line no 59: to be used as the initial diagnostic test for pulmonary TB in adults.

Thus, I suggest acceptance of this article, as this study is helpful for the stakeholders to make a right decision about a launch and roll-out of new TB diagnostics.
